# Tetanus Toxoid Vaccination Uptake and Associated Factors among Mothers Who Gave Birth in the Last 12 Months in Errer District, Somali Regional State, Eastern Ethiopia

**DOI:** 10.1155/2020/4023031

**Published:** 2020-05-07

**Authors:** Tsige Shushay Gebremedhin, Fissaha Tekulu Welay, Meresa Berwo Mengesha, Natnael Etsay Assefa, Weldu Mammo Werid

**Affiliations:** ^1^Department of Public Health, College of Medicine and Health Science, Adigrat University, Adigrat, Ethiopia; ^2^Departments of Midwifery, College of Medicine and Health Science, Adigrat University, Adigrat, Ethiopia

## Abstract

**Background:**

Maternal tetanus is defined as tetanus acquired during pregnancy or within 6 weeks after the end of conception. As tetanus is a vaccine-preventable disease, immunization of pregnant mothers with a TT (tetanus toxoid) dose is one of the most effective ways to protect against the disease. Some studies showed that 94% of neonatal mortality reduction could be achieved through immunization of pregnant and childbearing-age mothers with at least two doses of TT vaccination.

**Objective:**

To assess the uptake of tetanus toxoid vaccine and associated factors among mothers who gave birth in the last 12 months in Errer district, Somali Regional State, Eastern Ethiopia, 2017.

**Methods and Materials:**

A community-based cross-sectional study design was implemented to study 440 mothers who gave birth in the last 12 months. Participants were selected using the strata and systematic sampling technique after conducting a preliminary survey. Data were collected through a face-to-face interviewer-administered questionnaire. The collected data was entered into EpiData version 3.02 and then exported to Statistical Package for the Social Sciences (SPSS) version 20. Bivariate and multivariate logistic regressions were carried out to see the association between variables at *p* < 0.05 and 95% confidence interval. Finally, the information was presented by using frequencies, summary measures, and tables.

**Result:**

The overall tetanus vaccination uptake (≥TT2) doses was found to be 51.8%, 95% CI (47.7%, 56.4%). The total number of mothers who complete the five TT doses was 31 (14.8%). Urban residence [AOR = 6.1, 95% CI: (2.33, 10.43)], multiparity [AOR = 2.3, 95% CI: (1.7, 6.4)], and traveling less than 30 minutes from the home to a health facility [AOR = 4.6, 95% CI: (1.34, 6.72)] were some the factors that were significantly associated with tetanus toxoid vaccination uptake. *Conclusion and Recommendation*. Although TT immunization is a scientifically proven mechanism to protect against maternal and neonatal tetanus, only half of the district mothers received ≥TT2 doses. Besides, our study revealed that the low vaccine uptake is attributed to long distance travel to reach a health facility, maternal illiteracy, and pastoralist lifestyle of mothers in the district. Thus, the regional stakeholders are required to scale up efforts on mother's awareness creation towards the importance of the vaccine through health education and to arrange outreach TT vaccination campaigns in distant pastoralist communities within the region.

## 1. Background

Maternal tetanus is defined as tetanus acquired during pregnancy or within 6 weeks after the end of conception (whether pregnancy ended with live birth, miscarriage, or abortion) [1]. As tetanus is a vaccine-preventable disease, immunization of pregnant mothers with the TT dose is one of the most effective ways to protect against the disease. The mothers who are fully vaccinated with TT doses develop protective antibodies against tetanus for about 3, 5, 10, and 30 years. Besides, the vaccination provides enormous protection against neonatal tetanus for three months postpartum [2]. Some studies showed that 94% of neonatal mortality reduction could be achieved through immunization of pregnant and childbearing-age mothers with at least two doses of TT vaccination. That is why TT vaccines are recommended to eliminate maternal and neonatal tetanus for these parts of the community [3, 4]. Neonatal tetanus shared the highest number of mortalities from the 3.3 million newborn deaths which occurred each year globally [3]. The World Health Organization (WHO) 2015 report showed that an estimated 34,019 newborn deaths occur due to neonatal tetanus [5]. Globally, twenty-two countries had not eliminated maternal and neonatal tetanus (MNT) yet, and 40 million pregnant mothers remained in need of immunization against maternal tetanus. The number of mothers living in high-risk areas and protected with at least two doses of TT vaccination during the 1999-2006 supplementary immunization activities was only 73 million [6]. Nowadays, TT2+ immunization coverage among pregnant mothers accounts for 75% worldwide ranging from 95% in South East Asia to 53% in the East Mediterranean and 63% in Africa [7]. Maternal tetanus continues to occur among mothers living in developing countries as labor and delivery occur at home without trained birth attendants. Thus, the unclean surface, with unclean hands and instruments, escalates the chances of acquiring the disease [8]. The results from different studies revealed a number of factors contributing to TT2+ utilization among mothers. According to the study conducted in India, educated and mothers in the age group of 20-30 had better uptake [9]. Furthermore, other studies showed that occupational status, maternal age, number of visits to health care facilities, distance from health facilities, and family size could also considerably alter TT immunization uptake [10–12]. On the contrary, the study from two places in Pakistan (Lahore and Peshawar) presents some reasons for not taking the vaccine such as long distance to reach a health facility, the myth that the vaccine is useless, harmful to the fetus, lack of awareness, fear of side effect, and sterility were among the mentioned reasons [13, 14].

Ethiopia has the highest maternal and neonatal tetanus morbidity and mortality rates in the world due to low TT immunization coverage. The Ethiopia Demographic Health Survey (EDHS) report of 2011 showed that only 48% of mothers were protected (get vaccinated TT2+) against tetanus at birth. A very static progress had been shown up to 2016 evidenced by only 49% of mothers who were protected at birth [15, 16]. This low utilization emerges as numerous deliveries occur at home with unsanitary conditions. Despite the country's effort of interventional policy to meet the WHO goals towards Maternal Newborn Tetanus Elimination (MNTE) through extended immunizations and campaigning tetanus toxoid vaccinations, Ethiopia continues to have the highest neonatal tetanus morbidity and mortality rates [16]. The problem worsens more extensively in pastoralist communities like the Somali region where mothers lead their lives by raising livestock and lack access to health care services due to distance, myth, and lack of awareness towards the vaccine. Thus, knowing the reliable estimate of tetanus toxoid vaccination uptake and factors affecting utilization is found to be very crucial to plan and implement a corrective intervention in Errer district of the Somali region of Ethiopia. Henceforth, this study is aimed at determining the status and predictors of TT vaccination in the study area.

## 2. Methods and Materials

### 2.1. Study Area, Design, and Population

This study was conducted in Errer district of the Somali Regional State located in the eastern part of Ethiopia from February 23 to March 23, 2017. A community-based cross-sectional study design was implemented to study 440 mothers. The district has a total population of 100,425 people. The majority of the people (75–80%) have a pastoral livelihood, and they use four health centers, seventeen health posts, and five private pharmacies. Thus, systematically selected mothers who gave birth in the last 12 months and lived in the randomly selected kebeles of the district were included in the study.

### 2.2. Study Samples and Sampling Procedure

The study participants were pregnant and childbearing-age (15-49) mothers. Although the highest number of samples was calculated from the second objective, the calculation has been made for both the first and second objectives. Hence, the number of samples was calculated using a double population proportion formula, using the Stat calculation of Epi Info statistical software version 7. The calculation assumes 95% confidence interval, 80% power, 5% margin of error, and the ratio of unexposed (60% uneducated) to exposed (79% educated mothers) almost equivalent to 1 [17]. Hence, the total sample calculated was 408; adding 10% contingency for the nonresponse rate, 449 mothers were planned for recruitment as a sample.

A stratified sampling technique was used to select study participants after the district with 15 kebeles is stratified into two (12 rural and 3 urban) kebeles. Then, using the lottery method, four from rural (Hurso, Germam, Halshu, and Dimtu) and one randomly selected kebele from urban were included in the study. Finally, a preliminary survey was conducted by health extension workers to register the number of eligible mothers from the selected kebeles. The total sample size was proportionally allocated to the selected kebeles, and a systematic random sampling method was used to get the study subjects based on their registration list.

### 2.3. Data Collection Tool and Procedure

The data collection tool was a pretested structured questionnaire adapted from EDHS 2011, by incorporating variables from different literatures. Hence, it contains sociodemographic characteristics, obstetric characteristics, knowledge of the mother towards the vaccine, and accessibility to a health facility. The questionnaires were translated from English to Somali and Amharic languages for the ease of data collection. Eight health extension workers and two diploma nurses were recruited as data collectors and supervisors, respectively. The data collection method was through a face-to-face interview. The respondents were contacted through home-to-home visits. If two or more mothers were living in a single household, one woman was selected randomly.

### 2.4. Data Quality Control

The principal investigator provides two days of training for data collectors and supervisors regarding the approach and components of the questionnaire. The familiarity, reliability, and internal validity of the tool had been proven by conducting a pretest on 5% of the total sample size in Jigjiga town. The completeness of each questionnaire was checked by the principal investigator and the supervisors on a daily basis. Double data entry was done by two data clerks, and consistency of the entered data was crosschecked by comparing the two separately entered data on EpiData.

### 2.5. Operational Definitions

Uptake was measured if the mother received ≥TT2 doses as the first dose is only used for the sake of boosting immunity.

Vaccinated by card only is when the mother had doses documented on the immunization card [18]. Vaccinated by card plus history is when both documentation on the immunization card of the mother and doses reported by the mother were considered (18).

Knowledgeability is measured the respondents correctly answer >3 out of 5 knowledge questions about tetanus toxoid vaccination [19].

### 2.6. Data Processing, Analysis, and Ethics

The data were first coded, entered, and cleaned using EpiData statistical software version 3.02 and then exported into SPSS statistical software version 20 for analysis. Descriptive statistical analysis such as simple frequencies, measures of central tendency, and measures of variability was used to describe the variables. Bivariate analysis was used to see the association between each independent variable and the outcome variable by using binary logistic regression. All variables with *p* value ≤ 0.25 were taken into the multivariable model to control for all possible confounders. Multi colinearity test was carried out to see the correlation between independent variables using a variable inflation factor (VIF), and one of the independent variables was dropped for those with VIF of >10. Finally adjusted odds ratio along with 95% CI was estimated to identify factors affecting tetanus toxoid vaccination uptake. The level of statistical significance was declared at *p* value < 0.05. Ethical clearance was secured by Haramaya University Institutional Health Research Ethics Review Committee (IHRERC). Informed written and signed consent was obtained from each participant after explaining the purpose and benefits of the study.

## 3. Result

### 3.1. Respondent's Sociodemographic Characteristics

A total of 440 mothers participated in the study with a response rate of 98%. The median (±SD) age of the mothers was 28 (±6.2) years. About 430 (97.7%) of them were married and 187 (42.5%) of them took some education. Majority (434, 98.6%) of the mothers were Muslim in religion, and 347 (79.1%) were Somali by ethnicity (Table 1).

### 3.2. Accessibility to the Health Facility

Out of 440 mothers, nearly half of the mothers (226, 51.4%) spent more than one hour to reach a nearby health facility from their home, while only 89 (20.2%) of the women traveled less than 30 minutes to reach the district's health facilities.

### 3.3. Obstetric Characteristics

The study assesses the parity status of the mothers. Hence, 94 (213%) mothers had one, 243 (55.2%) two to four, and 103 (23.4%) five and above birth orders. Beside, 372 (84.5%) mothers had future fertility intentions, and 248 (56.4%) mothers had an ANC visit during their last pregnancy comprising 223 (89.9%) who had <4 and 25 (10.1%) who had ≥4 times ANC follow-up.

### 3.4. Knowledge of Mothers about TT Vaccine

Majority (360, 81.8%) of the mothers stated the purpose of tetanus toxoid vaccination. The number of mothers who satisfy knowledge measurement towards the vaccine was 213 (48.4%), mentioning three and above out of five scales of measurement. The least correctly mentioned question during the assessment of knowledge was the total number of TT doses the women were expected to receive in their lifetimes. Thus, only a few (55, 12.5%) mothers responded correctly stating five times (Table 2).

### 3.5. Tetanus Toxoid Vaccination Uptake

Regarding TT vaccination, 278 (63.2%) mothers were vaccinated for any type of TT dose during their latest pregnancy. However, vaccination uptake is considered when a mother received ≥TT2 doses, which accounts for 228 (51.8%), 95% CI (47.7%, 56.4%), mothers in this study. Meanwhile, only 31 (7%) mothers completed the recommended five TT doses. The study also showed that 65 (14.8%) mothers had TT vaccination after birth in the last 12 months (Table 3).

### 3.6. Factors Associated with the Uptake of Tetanus Toxoid Vaccination

In bivariate logistic regression, nine variables showed an association with TT vaccination status at a *p* value of ≤0.25, whereas the multivariate analysis revealed that urban residency, short distance travel, and maternal and paternal education had an association with ≥TT2 dose uptake (Table 4).

## 4. Discussion

Tetanus toxoid vaccination has enormous health benefits for both the mother and the newborn. TT vaccination uptake ≥ TT2 doses is considered to have a significant period of protection against tetanus infection. The study found that the uptake of two and above tetanus toxoid vaccinations among mothers accounts for 51.8% (95% (47.7, 56.4)) which comprises (22.3%) TT2, (15.2%) TT3, (7.3%) TT4, and (7%) TT5. This was lower than the result found from the Lahore and Peshawar districts of Pakistan [20] and the town Ambo in Ethiopia [18]. The difference might be explained as the pastoralist community lacks access to health care services and had poor health-seeking behavior. But unfortunately, the uptake was found higher for TT1 in our study; this is because most of the mothers who received the first dose were due to visiting the health facility for other services other than taking the vaccine. The subsequent doses would be discontinued once their initial health problems got relieved. The results from areas hard to reach like Lago metropolis showed that vaccine uptake is higher for TT1 and TT2, but lower for TT3 to TT5 [21] compared with our study. This difference might be because the reference study was conducted only in rural areas, where TT1 and TT2 doses were received through outreach during pregnancy and there is a lack of awareness to continue taking the doses after delivery. The demographic health survey report of Somaliland [22] and results from a study conducted in Elaramash, rural areas of Sudan in 2008 [23], revealed that TT vaccine uptake was slightly higher than our study. Thus, large surveys are known for their inclusive nature in terms of sample size from improved health care delivery system, since our study only considered mothers who delivered in a specific district. The result of our study showed similarities in TT dose uptake with the study conducted in Vientiane district of Lao PDR [24]. Our study showed that urban residents were more likely to vaccinate for TT2 and the above doses as compared to rural residents. This is in line with the result from Peshawar, Pakistan, and Tselemti district of the Tigray region of Ethiopia [14, 17]. This agreement might arise from mothers who lived in urban areas who had better access to information and a health facility. Hence, this helped the mothers to utilize the services compared to rural residents. Our study showed that maternal education enhances a mother's decision to vaccinate for tetanus toxoid, which is supported by a similar study from Peshawar and Lahore districts of Pakistan, Tselemti district of Tigray, and Jhapa district of Nepal [14, 17, 20].

The overall time spent to reach the health facility determines vaccination uptake of TT doses that is also supported by our study and results from Peshawar and Ambo [14, 18]. Hence, mothers who traveled less than 30 minutes to reach a health facility had better vaccination uptake compared to those who traveled more than one hour. Our study revealed that having ANC follow-up during pregnancy would help mothers' uptake of TT2 and above doses (*p* < 0.005). This implied that taking the ANC service was one of the entry points to get maternal TT vaccination. Meanwhile, prior knowledge of mothers about the vaccine provides an enormous advantage to receive the doses, both in our study and the study conducted in the Luanshya district of Zambia [19]. Hence, knowledge increases the health-seeking behavior of the mother and opens an opportunity to vaccinate for TT doses during childbearing age.

## 5. Conclusion and Recommendations

Although TT immunization is a scientifically proven mechanism to protect against maternal and neonatal tetanus, only half of the district mothers received ≥TT2 doses. Besides, our study revealed that low vaccine uptake is attributed to long-distance travel to reach a health facility, maternal illiteracy, and pastoralist lifestyle of the region. Thus, Somali regional health officials in collaboration with the Wereda health policy implementing body should scale up the mother's awareness towards the importance of the vaccine through health education, arranging outreach TT vaccination campaigns in distant pastoralist communities within the region. Furthermore, more studies are needed to determine the absolute proportion of mothers protected from tetanus at birth by antibody testing.

## 6. Limitation of the Study

As this study used a cross-sectional study design, it is weak in showing temporal relationship b/n cause and effect. In addition, the inclusion of mothers up to 12 months postpartum may result in some recall bias given that mothers took the vaccine at the time of labor and delivery and may be confused about the type of vaccine they took as other therapeutic drugs like oxytocin may have been given in the same period.

## Figures and Tables

**Table 1:** Sociodemographic characteristic of mothers who gave birth in the last 12 months in Errer district, Somali Regional State, Eastern Ethiopia, March 2017 (*N* = 440).

Variables		Frequency	Percent
Residence	Urban	92	20.9
	Rural	348	71.1

Maternal age	15-19	28	6.4
	20-34	340	77.3
	35-49	72	16.3

Family size	1-3	101	23
	4-6	205	46.5
	≥7	134	30.5

Ethnicity	Somali	348	79.1
	Oromo	86	19.5
	Amara	6	1.4

Maternal level of education	No education	253	57.5
	Some education	187	

Husband level education	No education	208	47.2
	Some education	232	52.8

Maternal occupation	Housewife	336	76.4
	Government employee	29	6.6
	Merchant	44	10
	Pastoralist	31	7

Husband occupation	Daily labor	33	7.5
	Farmer	88	20
	Pastoralist	206	46.8
	Government employee	113	25.7

**Table 2:** Knowledge of TT vaccine among mothers who gave birth in the last 12 months in Errer district, Ethiopian Somali Regional State, March in 2017 (*N* = 440).

Variables	Frequency	Percent
Knowledge of TT vaccine		
A vaccine giving to women of childbearing age	325	73.9
Family planning provided to women	8	1.8
I do not know	107	24.3
Eligible for TT vaccine		
All childbearing-age women including 15 years and above school girls	61	13.9
Only infants	39	8.9
Only pregnant women	195	44.2
I do not know	145	33.0
Appointment from 1^st^ dose to 2^nd^ dose of TT vaccine		
After one week	4	1.0
After four weeks	236	53.6
After six weeks	31	7.0
After one year	11	2.5
I do not know	158	35.9
Total number of TT vaccines women should receive in their lifetime		
Two times	8	1.8
Three times	30	6.8
Four times	20	4.5
Five times	55	12.5
I do not know	327	74.4
Purpose of tetanus toxoid vaccine		
To protect only the mother from tetanus	10	2.3
To protect both the mother and the baby from tetanus	360	81.8
To prevent pregnancy	4	9

**Table 3:** TT vaccination characteristics of mothers who gave birth in the last 12 months in Errer district, Somali Regional State, Eastern Ethiopia, in March 2017.

Variables		Frequency	Percent
TT vaccination in the last pregnancy (*N* = 440)	Yes	278	63.2
	No	162	36.8

Evidence of TT vaccination (*n* = 278)	Card	65	23.4
	History (oral)	113	40.6
	Both	100	36.0

Place of TT vaccine uptake (*n* = 278)	Health post	123	44.2
	Health center	115	41.4
	Hospital	10	3.6
	Home	30	10.8

TT vaccination after birth in the last 12 months (*N* = 440)	Yes	65	14.8
	No	375	85.2

Current TT vaccination status (*N* = 440)	Not vaccinated	60	13.6
	TT1	152	34.5
	TT2	98	22.3
	TT3	67	15.2
	TT4	32	7.3
	TT5 and above	31	7.0

TT vaccination uptake (*N* = 440)	≥TT2	228	51.8
	<TT2	212	48.2

**Table 4:** Factors associated with the uptake of tetanus toxoid vaccination among mothers who gave birth in the last 12 months in Errer district, Somali Regional State, Eastern Ethiopia, in March 2017.

Variables	Frequency (5%)	Tetanus toxoid uptake		COR (95% CI)	AOR (95% CI)
		≥TT2	<TT2		
Maternal education					
Some education	187 (42.5%)	160	27	4.2 (2.1, 6.3)^∗∗^	2.16 (1.4, 4.8)
No education	253 (57.5%)	68	185	1.00	1.00
Husbands education					
Some education	232 (52.8%)	171	61	3.4 (2.01, 7.6)^∗∗^	2.02 (1.4, 8.2)
No education	208 (47.2%)	57	151	1.00	1.00
Maternal occupation					
Housewife	336 (76.4%)	148	188	1.00	1.00
Government employee	29 (6.6%)	26	3	6.5 (2.4, 9.8)^∗^	2.5(1.6, 10.05)
Merchants	44 (10%)	33	11	2.4 (1.05, 8.51)	0.8 (0.04, 1.01)
Pastoralist	31 (7%)	21	10	0.51 (0.02, 0.9)	0.14 (0.001, 0.5)
Husband occupation					
Daily labor	33 (7.5%)	30	3	1.00	1.00
Farmers	88 (20%)	58	30	0.12 (0.04, 0.71)	0.01 (0.002, 1.2)
Pastoralist	206 (46.8%)	57	149	0.03 (0.002, 0.07)	0.001 (0.004, 0.02)
Government employee	113 (25.7%)	83	30	1.4 (0.9, 3.9)^∗^	1.25 (0.2, 5.34)
Residence					
Urban	92 (20.9%)	83	9	6.1 (2.33, 10.43)^∗∗^	4.3 (1.6, 13.21)^∗^
Rural	348 (79.1%)	147	201	1.00	1.00
Parity					
Primi- (1)	94 (21.3%)	51	43	1.4 (1.05, 3.04)^∗^	1.2 (0.04, 4.5)
Multi- (2-4)	243 (55.2%)	138	105	3.1 (1.6, 6.3)^∗∗^	2.3 (1.7, 6.4)
Grand multi- (≥5)	105 (23.4%)	39	64	1	1
Time taken from home to health facility					
<30 minutes	89 (20.2%)	80	9	7.1 (3.1, 11.71)^∗∗^	4.6 (1.34, 6.72)^∗^
30 min-1 hr.	124 (28.2%)	102	22	1.5 (0.9, 5.34)^∗^	0.8 (0.26, 5.9)
More than one hr.	227 (51.6%)	47	180	1.00	1.00
ANC follow-up					
Yes	248 (56.4%)	198	50	8.3 (4.34, 15.92)^∗∗^	5.4 (2.8, 10.9)^∗^
No	192 (43.6%)	32	160	1.00	1.00
Knowledge of the mother					
Knowledgeable	213 (48.4%)	173	40	7.4 (3.9, 13.67)^∗^	3.45 (1.89, 9.56)^∗^
Not knowledgeable	227 (51.6%)	57	170	1.00	1.00

CI: confidence interval; AOR: adjusted odds ratio; COR: crude odds ratio. ^∗^*p* < 0.05; ^∗∗^*p* < 0.001.

## Data Availability

The data used to support the findings of this study are available from the corresponding author upon request.

## Supplementary Materials

Supplementary Materials This is the data set from which we extract the result in the study.
